# Enzyme-Assisted Amplification and Copper Nanocluster Fluorescence Signal-Based Method for miRNA-122 Detection

**DOI:** 10.3390/bios13090854

**Published:** 2023-08-28

**Authors:** Yang Qing, Haobin Fang, Yuxing Yang, Yazhen Liao, Haiyu Li, Zhencui Wang, Jie Du

**Affiliations:** State Key Laboratory of Marine Resource Utilization in South China Sea, College of Materials Science and Engineering, Hainan University, Haikou 570228, China; 20080500210023@hainanu.edu.cn (Y.Q.); 21220856000011@hainanu.edu.cn (H.F.); 20085600210065@hainanu.edu.cn (Y.Y.); 21220856000036@hainanu.edu.cn (Y.L.); 20080500110012@hainanu.edu.cn (H.L.); 20080500110014@hainanu.edu.cn (Z.W.)

**Keywords:** exonuclease III, miRNA-122, CuNCs, fluorescence reduction

## Abstract

At present, a large number of studies have demonstrated that miRNAs can be used as biological indicators for the diagnosis and treatment of diseases such as tumours and cancer, so it is important to develop a new miRNA detection platform. In this work, miRNA-122 is used as the basis for targeting detection agents. We have designed an unlabelled DNA1 that undergoes partial hybridisation and has a 20 T base long strand. The fluorescent signal in this experiment is derived from copper nanoclusters (CuNCs) generated on the circular T-long strand of DNA1. At the same time, DNA1 is able to react with miRNA-122 and achieve hydrolysis of the part bound to miRNA-122 via the action of nucleic acid exonuclease III (Exo III), leaving a part of the DNA, called DNA3, while releasing miRNA-122 to participate in the next reaction, thus achieving circular amplification. DNA3 is able to react with DNA2, which is bound to streptavidin magnetic beads (SIBs) and separated from the reaction solution via the application of a magnetic field. Overall, this is a fluorescence signal reduction experiment, and the strength of the fluorescence signal from the copper nanoclusters can determine whether the target miRNA-122 is present or not. The degree of fluorescence reduction indicates how much DNA1, and thus the amount of target miRNA-122, has been hydrolysed. By evaluating the variations in the fluorescence signal under optimised conditions, we discovered that this method has good sensitivity, with a detection limit as low as 0.46 nM, better than many other previous works on fluorescence signal-based biosensors for miRNA detection. This technique offers high discrimination and selectivity and can serve as a persuasive reference for early diagnosis.

## 1. Introduction

MicroRNAs (miRNAs) are an important class of endogenous non-coding small RNA molecules, generally consisting of 20–24 nucleotides of non-coding RNA molecules [1], which are widely used to regulate gene expression in animals and plants [2], replication and regulation of viruses [3], etc. After the discovery of the first miRNA in 1993 [4], more than three thousand miRNAs have been identified in plants and animals, and the latest study inferred 2300 true human mature miRNAs using in silico and experimental low-throughput validation strategies [5], indicating that miRNAs play a very important role in living organisms. Different miRNAs regulate different types of genes; miRNA-122, for example, accounts for 70% of the total amount of miRNAs in the liver [6]. MiRNA-122 was discovered to play several roles in the pathologies of acute liver failure (ALF) [7], alcoholic liver disease (ALD) [8], hepatitis C virus (HCV) [9], and hepatocellular carcinoma (HCC) [10]. Recent pathological investigations have revealed that differing levels of miR-122 expression in HCC might contribute to tumour metastasis and development [11], which can impede growth, cause metastasis, and even impair angiogenic activity in HCC cells [12]. As a result, a series of biosensors are required to realise the changes and monitoring of micRNA, and hence the prevention and treatment of disease [13].

Based on their simplicity, high specificity, and rapid detection, many researchers have chosen to use fluorescent detection methods and biosensors, and many research results have been obtained thereby [14]. In recent years, most amplification strategies, such as loop-mediated isothermal amplification (LAMP) [15], catalytic hairpin assembly (CHA) [16], polymerase chain reaction (PCR) [17], and hybridisation chain reaction (HCR) [18], have been successfully applied to miRNA detection. Meanwhile, breakthroughs in nanotechnology have had a significant impact on the field of biosensors [19]. A large amount of research in the exploration and synthesis of various types of nanomaterials has long been used for subsequent fluorescent biosensors [20]. For example, since the first report on the formation of fluorescent copper nanoclusters was presented in 1998 [21], the clusters are now capable of detecting various types of targets ranging from ions to macromolecules, such as the determination of iron [22], the detection of lymphophilic viral DNA [23], the detection of E. coli [24], etc. Meanwhile, compared to noble metals, copper nanoclusters are not only more accessible and cheaper but also exhibit unique photoluminescence properties with large Stokes shifts, low toxicity, and high biocompatibility [25]. Moreover, the fluorescence properties of CuNCs depend on their nanometre size and can be modulated by playing with different synthesis conditions [26]; e.g., they show different fluorescence properties on single-stranded DNA [27] and double-stranded DNA [28]. The fluorescence is also restricted to nanoclusters with fewer than 10 copper atoms, obtained using very low stoichiometric amounts of reducing agent relative to copper (α < 0.1) [29]. Ma and colleagues [30] described a simple enzyme-free and label-free fluorescence biosensor for the quantitative detection of let-7a.To establish a fluorescent biosensor, the distance-dependent properties of photo-induced electron transfer (PET) between G-quadruplex/haemin (G/haemin) complexes and fluorescent DNA/Cu nanoclusters were observed by monitoring the formation of DNA/CuNCs.

Here, we designed a copper nanocluster with fluorescent properties based on the specific hydrolysis of DNA strands with Exo III [31], the generation of copper nanoclusters with fluorescent properties on the long strand of the hairpin structure rather than on the straight strand T alone [32], and the magnetic separation of streptavidin immunomagnetic beads (SIBs) [33], for the detection of miRNA-122 as a fluorescent biosensor. In the first step, the target material miRNA-122 is allowed to react with DNA1 with a T long-chain circular hairpin structure, and hydrolysis is achieved via the action of Exo III, releasing the DNA3 fragment; in the second step, a DNA2 solution with streptavidin immunomagnetic beads attached is added, reacted, and separated under the conditions of an applied magnetic field; in the third step, appropriate amounts of copper sulphate and sodium ascorbate solution are added to the separated solution to carry out the reaction. Finally, a fluorescence assay was performed to achieve the detection of the target material miRNA-122 via a reduction in fluorescence value. The sensitivity and specificity of the designed DNA nanobiosensor without fluorescent labelling were measured.

## 2. Materials and Methods

### 2.1. Materials

All chemicals and reagents were analytical reagent-grade, used as received without further purification. Copper sulphate pentahydrate was purchased from McLean Biochemical Co., Ltd. (Shanghai, China). pH reference solution (4M LiCl) was purchased from Merrill Chemicals Technology Co., Ltd. (Shanghai, China). Sodium chloride (NaCl) was purchased from Aladdin Co., Ltd. (Shanghai, China). Tween-20 was purchased from KGI Biotechnology Co., Ltd. (Jiangsu, China). Sodium ascorbate was purchased from Solaibao Technology Co., Ltd. (Beijing, China). Ultrapure water was purchased from Myriad Chemical Technology Co. (Beijing, China). Nucleic acid exonuclease III (ExoIII) and NEbuffer 1 were purchased from New England Biolabs (Guangzhou, China). Streptavidin immunomagnetic beads (SIBs) and PBS (10×) were purchased from Biotech Bioengineering Co. (Shanghai, China). All DNA oligomers and miRNAs were purchased from Bioengineering Co., Ltd. (Shanghai, China), listed in Table S1. The 3’ end of DNA2 was labelled by Biotin.

### 2.2. Apparatus

Fluorescence signal values were measured via RF-6000 fluorescence spectrophotometer (Shimadzu, Kyoto, Japan) in this experiment. Copper nanoclusters generated on the long-strand T-base of DNA1 were used as the fluorescence signal. A centrifuge (D2012, SCILOGEX Corporation, Rocky Hill, CT, USA) was used for the separation and purification of nano-DNA after ligation.

### 2.3. Preparation of Reagents

DNA was diluted to 100 mol L^−1^ with ultrapure water (enzyme-free, sterile), and miRNA was diluted to 20 mol L^−1^ with DEPC-treated water. The buffer used in the experiment was PBS buffer (1×) stored in a 4 °C refrigerator. Exo III was kept at −20 °C and configured to 2 U/µL for use. The TTL buffer consisted of 1 M LiCl solution, 100 mM Tris, and 0.1% Tween-20, kept at 4 °C. Other reagents were added according to their instructions.

### 2.4. Generation of CuNCs

The CuNCs using the PolyT template were synthesized according to the previous work [32]. The experiments were performed in 200 μL 1× phosphate buffer solution containing 0.5 µM probe DNA, 250 µM copper sulphate, and 6 mM ascorbic acid, completely mixed and incubated at 37 °C for 10 min to form fluorescent CuNCs.

### 2.5. Streptavidin Immunomagnetic Beads Functionalisation

Streptavidin immunomagnetic beads (SIBs) were functionalized according to Wang et al. [34]. First, they were washed twice with 1 mL of TTL buffer and once with PBS. Then, they were annealed with 1.5 µL of DNA-c and cooled to room temperature. Next, to them were added 1 µL of magnetic beads, which reacted at 37 °C for 1 h. At the end of the reaction, the magnetic beads were washed twice with PBS buffer (1×, pH = 7.4) and suspended in 90 µL of PBS to obtain SIBs-DNA. All washing steps were carried out under an external magnetic field.

### 2.6. Detection of Target miRNA-122

Diluted reaction solutions of different specific concentrations were added to 0.5 mL microtubes. First, 47 μL of PBS (1×) and 1 μL of DNA1 (100 μM) were added to the centrifuge tube, annealed at 95 °C, and slowly cooled to room temperature, then maintained at room temperature for at least 1 h to ensure that the substrate could form the desired hairpin structure. In the second step, 2 μL of Exo III (2 U/μL) and a certain amount of miRNA-122, which was to be detected, were added and reacted at 37 °C for 2 h, followed by a high-temperature reaction at 95 °C for 10 min to completely inactivate the Exo III. In the third step, 90 μL suspension of streptavidin immunomagnetic beads with DNA3 attached, including 1.5 μL of DNA3 (100 μM), 1 μL of streptavidin immunomagnetic beads, and PBS (1×), named SIBs-DNA3, were added and reacted at 37 °C for 1 h. In the fourth step, the above reaction solution was placed on a magnetic frame for 2 min and then separated. The remaining solution was added to 20 μL Cu_2_SO_4_ solution (2.5 mM) and 40 μL sodium ascorbate (30 mM), shaken well, then reacted at 37 °C for 10 min, and finally tested via fluorescence spectrophotometer.

### 2.7. Testing miRNA-122

The fluorescence signal values were measured via RF-6000 fluorescence spectrometer (Shimadzu, Japan) using a four-pass quartz cuvette with cover (10 mm). The test solution was 2 mL of ultrapure water plus the experimental solution. The fluorescence intensity was measured under conditions of 340 nm excitation and 671 nm emissions. The fluorescence was measured in the range of 550 nm to 750 nm, with a broadband excitation spectrum of 5 nm and a broadband emission spectrum of 10 nm.

## 3. Results and Discussion

### 3.1. Principle of miRNA Detection

**Figure 1:**
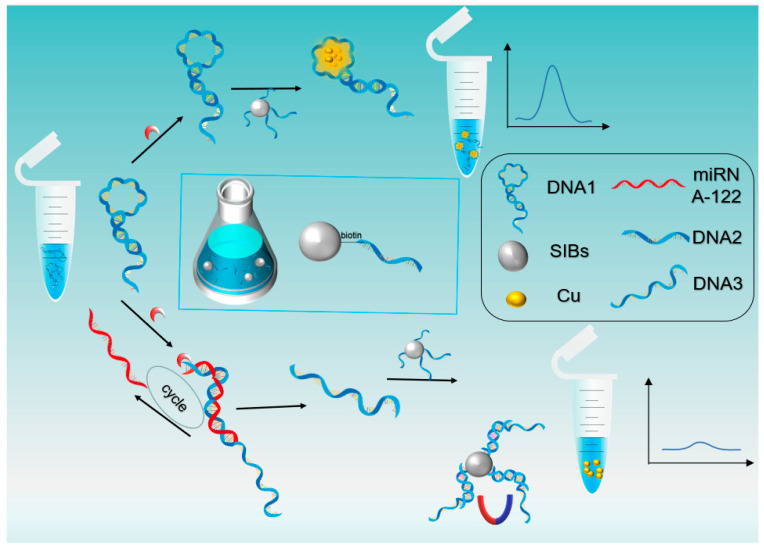
Principles of detection of biosensors based on enzyme-assisted amplification and closed DNA template copper nanocluster fluorescence signal for miRNA detection.

The analytical mechanism of this sensing system is shown in Figure 1. In this strategy, DNA1 with a hairpin structure of 20 T bases was designed specifically to achieve partial hybridisation of its own bases after annealing at 95 °C and ensure that the 3’ end is exposed, thus forming long-stranded T-loop DNA, and the long-stranded T-loop can be bound to copper nanoclusters to generate fluorescence. Meanwhile, one end of the long strand of DNA1 can undergo complete complementary base pairing with miRNA-122, which is hydrolysed under the action of Exo III from the 3’ end of DNA1, where hybridisation occurs, up to the T-base of the long strand that does not react with miRNA-122 and releases fragment DNA, named DNA3; meanwhile, the unhydrolysed miRNA-122 reacts with other DNA1 to trigger a 1:N cycle reaction, thus realizing the effect of enzyme-assisted amplification in order to detect the target material. Second, after the solution is inactivated by Exo III at a temperature of 95 °C, the residual DNA3 can partially hybridise with DNA2, which is attached with streptavidin immunomagnetic beads, by labelling biotin at the 3’ end and is separated from the original solution under the condition of an applied magnetic field. Finally, a certain amount of copper sulphate solution and sodium ascorbate solution are added to the original solution remaining after the separation of magnetic beads for reaction, and then fluorescence testing is performed. The TEM images of nanoclusters after interaction with miRNA-122 are shown in Figure S1C. Due to the reduction of T-base content in the solution, most of the reduced copper nanoclusters aggregate into large copper substrates and do not exhibit fluorescence properties, so the fluorescence values tested are low.

In the absence of the target material miRNA-122, the hairpin structure of DNA1 will not be opened and hydrolysed by Exo III, and the fragment DNA2, which can react with DNA3, will not be exposed. Therefore, DNA1 will not react with DNA3, and DNA1 will not be separated from the original solution under the applied magnetic field condition. Finally, after adding copper sulphate solution and sodium ascorbate solution to the unreacted solution of DNA1, nano-copper can form copper nanoclusters at the long chain T-loop and exhibit fluorescence properties with high fluorescence values tested. The TEM images of nanoclusters in the absence of miRNA-122 are shown in Figure S1A,B.

### 3.2. Feasibility Study of the miRNA-122 Assay

**Figure 2:**
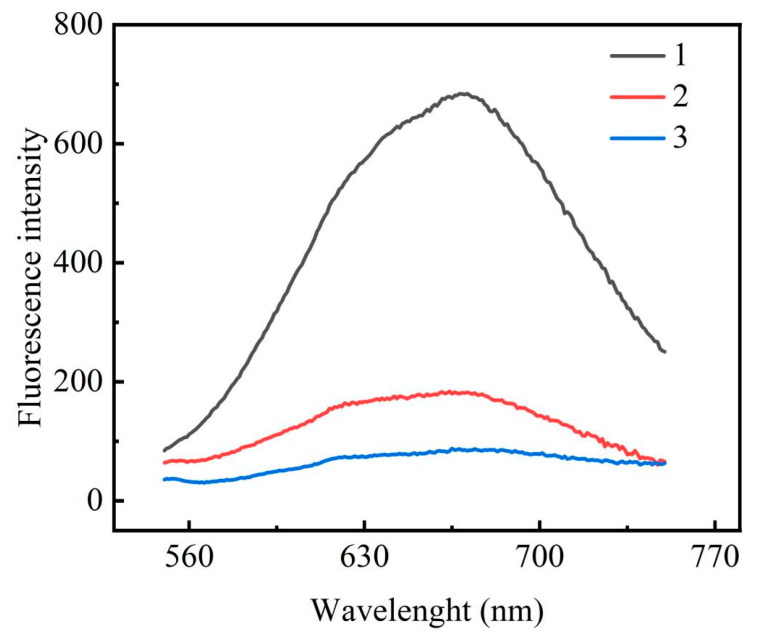
For feasibility analysis, curve 1 shows the system fluorescence without miRNA-122; curve 2 shows the system fluorescence in the absence of SIBs-DNA3; and curve 3 is the solid system fluorescence with 50 nM of miRNA-122.

The feasibility of the strategy was verified by examining the fluorescence response signal of the system before and after adding the detection target object. The experimental results are shown in Figure 2, with all the same test conditions. The black curve 1 in Figure 2 is the fluorescence curve measured in the absence of the target miRNA-122 and in the presence of SIBs-DNA2, which shows that copper nanoclusters were generated on the hairpin DNA1 at this time and showed obvious fluorescence intensity, and the comparison with curve 3 later allows the conclusion to be drawn that SIBs-DNA2 does not react with DNA1; the red curve 2 is the fluorescence curve measured in the presence of miRNA-122 and in the absence of SIBs-DNA2. Under 122 conditions and without the addition of SIBs-DNA2, the remaining DNA3 generated a certain fluorescence value after the addition of the copper sulphate solution and sodium ascorbate solution, and comparison with curve 1 indicates that the fluorescence value generated by the copper nanoclusters under the T-loop of DNA1 is much larger than that generated by the long chain T of DNA3, at which time DNA1 has been successfully hydrolysed by Exo III; blue curve 3 is the fluorescence value generated by the T-loop of DNA1 after the addition of miRNA-122 and SIBs-DNA2 under the conditions of the final experimental results, and comparison with curve 2 indicates that the fluorescence value significantly decreased, indicating that there is no T long chain remaining in the solution. The surface comparison with curve 1 indicates that DNA1 has been completely reacted in the experiment, suggesting that the target detectable miRNA-122 was involved in the above reaction. Therefore, the feasibility of this experiment can be verified from Figure 2.

### 3.3. Optimisation of Experimental Conditions

In this experiment, the successful generation of copper nanoclusters is an important step that affects the experiment. Therefore, it is necessary to evaluate the effective parameters that affect fluorescence intensity. In this study, factors such as copper concentration, reaction temperature, Exo III dosage, and hybridisation time were investigated, and other conditions, namely DNA1 (100 μM) dosage of 1 μL, miRNA-122 (20 μM) dosage of 0.5 μL, and DNA2 (100 μM) dosage of 1.5 μL, were kept constant while changing one variable. The results of the study were as follows:

#### 3.3.1. Optimisation of EXO III Reaction Conditions

**Figure 3:**
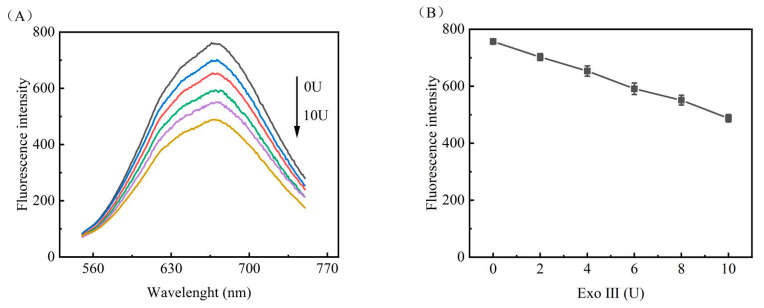
Analysis of the EXO III effects. The curve of (**A**) shows the addition of 0 U, 2 U, 4 U, 6 U, 8 U, and 10 U; (**B**) shows the dot plot corresponding to the highest fluorescence value of 340 nm excitation for each experiment three times.

In order to obtain optimal performance from the DNA fluorescent biosensor constructed in this experiment and to realise the best detection effect, a number of experimental conditions were optimised. Firstly, after the experiment, it was found that the amount of Exo III added in the enzyme-assisted cyclic amplification seriously affected the generation of copper nanoclusters. In order to explore the influence relationship, experimental groups of 6 microtubes were designed to add different volumes of nucleic acid exonuclease III (2 UμL^−1^) under the condition of no addition of miRNA-122 according to the procedure of this experiment; 0 U, 2 U, 4 U, 6 U, 8 U, and 10 U were added; and all experiments were tested three times. The experimental results are shown in Figure 3, and the fluorescence values decreased gradually with the increase of the amount of nucleic acid exonuclease III as shown in Figure 3A; the linear relationship in Figure 3B shows that the fluorescence values decreased by nearly one-third from 0 U to 10 U.

**Figure 4:**
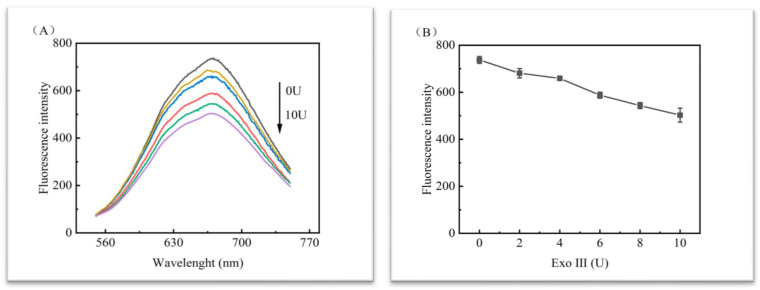
Analysis of the EXO III effects. The curves from top to bottom in (**A**) show the addition of 0 U, 2 U, 4 U, 6 U, 8 U, and 10 U of the already-inactivated enzyme; (**B**) shows the dot plot corresponding to the highest fluorescence value for 340 nm excitation.

In order to rule out the possibility that DNA1 was hydrolysed by Exo III, six more sets of experiments were set up under the conditions of adding different volumes of inactivated Exo III without adding miRNA-122, each set of experiments was tested three times with the same amount as above, and the experimental results were obtained in Figure 4. The addition of inactivated Exo III had the same effect on the fluorescence of the test; as the amount of Exo III added increased, the fluorescence value decreased and showed a certain regularity. Figure 3 and Figure 4 illustrate that Exo III, as a protein in the reaction solution, hinders the formation of copper nanoclusters on the DNA strand, thus leading to a decrease in fluorescence values.

**Figure 5:**
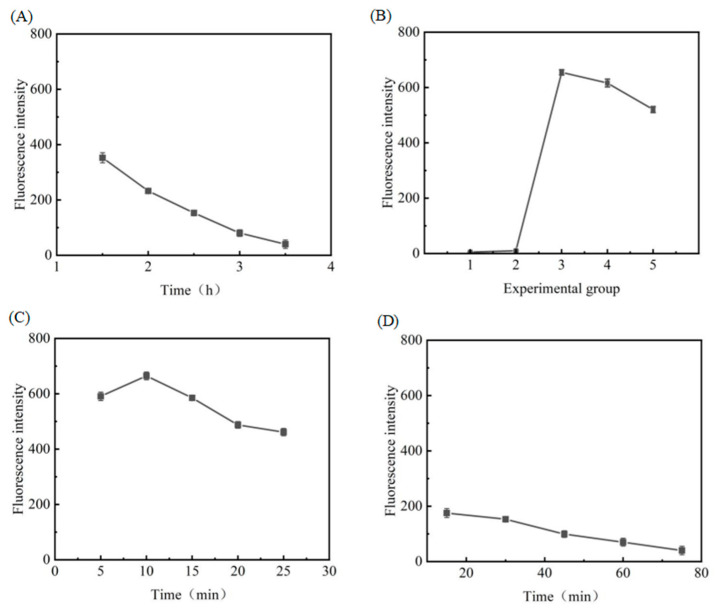
Optimisation of experimental conditions: (**A**) optimisation of reaction time of exonuclease; (**B**) optimisation of concentration of copper sulphate and sodium ascorbate; (**C**) optimisation of reaction time of copper nanometre; and (**D**) optimisation of reaction time between DNA3 and SIBs-DNA.

Therefore, considering the subsequent experimental requirements and the effect of Exo III, different from other experiments to optimise the enzyme concentration, the amount of enzyme added in this experiment was determined to be 4 U, and the enzyme reaction time was investigated and optimised. Specifically, when five sets of experiments were set up, all the added substances were the same. Only the enzyme reaction time was different, and the experimental results were obtained. As shown in Figure 5A, only the enzymatic amplification reaction time of Exo III was changed with other conditions unchanged, the experimental results showed that the fluorescence reduction stabilised after the reaction time reached 3 h, and the DNA1 in the surface reaction system was essentially completely reacted. Therefore, the reaction condition of 4U enzyme reaction for 3 h was chosen in the subsequent experiment.

#### 3.3.2. Optimisation of Copper Sulphate and Sodium Ascorbate Concentrations

In this experiment, the concentration of copper sulphate solution was 2.5 mM and the concentration of sodium ascorbate solution was 30 mM. In order to determine the best fluorescence value of copper nanoclusters generated under the influence of the same template DNA1 and the same concentration of Exo III, five groups of experiments were set up, numbered 1 (5 mL copper sulphate and 10 mL sodium ascorbate), 2 (10 mL copper sulphate and 20 mL sodium ascorbate), 3 (20 mL copper sulphate and 40 mL sodium ascorbate), 4 (30 mL copper sulphate and 60 mL sodium ascorbate), and 5 (40 mL copper sulphate and 80 mL sodium ascorbate), and tested three times, and the experimental results are shown in Figure 5B.

From Figure 5B, we can see that the best fluorescence values were obtained in the third group with 20 mL of copper sulphate and 40 mL of sodium ascorbate, and the basic lack of fluorescence in groups 1 and 2 also indicates that Exo III has an effect on the generation of copper nanoclusters, most likely due to the obstruction of the binding of copper atoms to DNA. The decrease in fluorescence values in groups 4 and 5 is due to the fact that the reduced copper concentration exceeds a certain value and aggregates itself, resulting in a decrease in the binding to DNA and a decrease in the concentration of copper nanocluster production exhibiting fluorescence properties. Therefore, the third set of experimental conditions, i.e., 20 mL of copper sulphate (2.5 mM) and 40 mL of sodium ascorbate (30 mM), were chosen as the subsequent experimental concentrations. The 4 U concentration of Exo III was also selected.

#### 3.3.3. Optimisation of Reaction Time of Copper Nanoclusters

The DNA used in this experiment was not labelled with a fluorescent tag, and the fluorescence tested was due to the reduction of copper ions in the background conditions of the long-stranded T-base to produce small-scale copper nanoclusters excited at 340 nm. Therefore, as small-sized fluorescent particles form, they may exhibit different fluorescence effects depending on the length of the reaction time. In order to distinguish the effect of time on the fluorescence values more accurately, five sets of experiments were designed with a total volume of 200 μL containing 1 U of DNA1 (100 μM), 4 U of Exo III, 20 mL of copper sulphate (2.5 mM), and 40 mL of sodium ascorbate (30 mM), as well as PBS buffer solution (1×). The reaction times were 5 min, 10 min, 15 min, 20 min and 25 min, respectively, and all experiments were tested three times. The experimental results are shown in Figure 5C. From Figure 5C, it can be seen that the fluorescence value tends to increase and then decrease with the increase of reaction time. The increase before 10 min is due to the increase of copper nanoclusters generated on DNA, the decrease after 10 min is due to the aggregation of the reduced copper with the increase of reaction time, and the fluorescence property will disappear when the aggregated copper exceeds a certain size. Therefore, we chose 10 min as the reaction time for the copper nanoclusters in this experiment.

#### 3.3.4. Optimisation of the Reaction Time of DNA3 with SIBs-DNA2

In order to prevent copper nanoclusters being generated from DNA3 left over from the DNA1 enzymatic amplification reaction and thus affecting the fluorescence values of the tested system, the experiment allowed DNA3 to react with SIBs-DNA2 and thus be completely separated from the system. Therefore, the reaction conditions of both were optimised. First, in order to reduce the experimental error and steps, the amount of DNA2 itself was allowed to be slightly more than the concentration of DNA1, so 1.3 μL of DNA2 (100 μM) was allowed to react with streptavidin immunomagnetic beads first, which theoretically ensured that DNA3 could completely undergo the hybridisation reaction. Next, the experimental reaction group was designed to optimise the reaction time of both. The experimental results are shown in Figure 5D. The fluorescence value was essentially unchanged after 60 min of the experimental results in both reactions, indicating that DNA3 was essentially separated from the system by the magnetic beads. Therefore, for the subsequent experiment, 60 min was chosen as the best reaction time.

### 3.4. Assay Performance for miRNA-122 Detection

Based on the above optimised experimental conditions, i.e., 4 U nucleic acid exonuclease III, 3 h enzymatic reaction time, 1 h reaction time with SIBs-DNA2, 20 mL copper sulphate (2.5 mM) and 40 mL sodium ascorbate (30 mM) added after the final separation, and a copper nanocluster reaction time of 10 min, the best results for fluorescence signal values could be obtained. Based on the standard procedure of this experiment, the assay system was used to detect different concentrations of target miRNA-122 to explore the sensitivity of the constructed assay to the target miRNA. Ten sets of experiments were set up to obtain the fluorescence intensity at different concentrations of miRNA-122, and each set of experiments was repeated three times.

**Figure 6:**
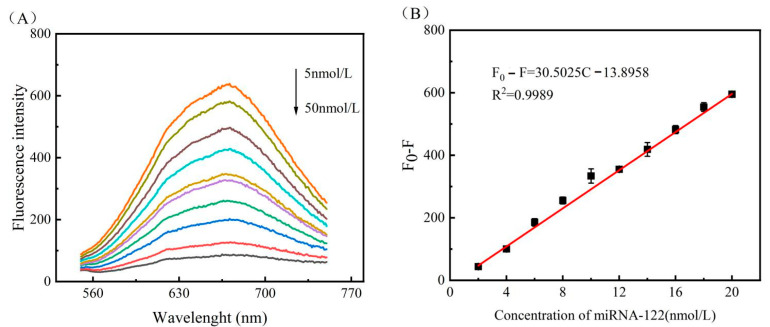
The signal response and standard detection curve of miR-122, where the concentration from top to bottom in (**A**) is 5 nM, 10 nM, 15 nM, 20 nM, 25 nM, 30 nM, 35 nM, 40 nM, 45 nM, and 50 nM, respectively; (**B**) shows linear regression of fluorescence intensity at λ = 671 nm with miR-122 concentrations. The error bars represent the standard deviation of three repeat experiments.

As shown in Figure 4A, from top to bottom, the fluorescence value decreases as the concentration of miRNA-122 increases. When the target detector concentration is as low as 5 nM, the fluorescence spectrum also shows a relatively obvious differentiation from the fluorescence spectrum without this detector (black curve 1 in Figure 3), indicating that this detection strategy can still show signal values at very low target concentrations, i.e., it has high sensitivity. Since this experiment yielded decreasing fluorescence values as the target miRNA concentration increased, the standard detection curve in the sensitivity analysis was obtained by subtracting the fluorescence value (F_0_) from the black curve 1 in Figure 3 for different concentrations (F) of miRNA-122, from 5 nM to 50 nM in Figure 6A. Ten different sets of data (F_0_−F) were obtained, and a linear regression curve F_0_ − F = 30.5025C − 13.8958 was plotted, where C represents the miRNA-122 concentration (nmol L^−1^). R^2^ = 0.9989 was obtained after a simple fit, indicating the high feasibility of this experiment, as shown in Figure 6B. The minimum limit of detection (LOD) was calculated based on the triple standard deviation (3σ) of the blank signal (without miRNA-122), and the limit of detection was 0.46 nM via simple calculation using the formula 3σ/S (where σ is the standard deviation of the blank signal and S is the slope of the fitted line). The above experimental results indicate that the sensing system can detect the target miRNA within the detection range with high accuracy, better than many previous works on fluorescence signal-based miRNA detection system, listed in Table 1.

### 3.5. Sensor Specificity and Reproducibility

The anti-interference performance of the detection platform is also an important indicator. To see the effect of other micro RNA sequences on the assay results, the signal values obtained from the detection of other homologous miRNAs similar to the target detectors were therefore examined under the same optimised conditions. Theoretically, the efficiency of other non-target miRNAs striving to carry out the next enzymatic reaction would decrease, and the same conditions would eventually lead to a small decrease in fluorescence intensity. In order to verify that the sensing system constructed in this experiment had the ideal selection ability for target miRNA-122, a variety of miRNAs were selected for specific detection experiments, including let-7a, miRNA-21, miRNA-141, miRNA-105, miRNA-205, miRNA-220, and miRNA-221. The concentration of the detectors in each group was 100 nM, except for the miRNA-122 group, where the concentration of the detectors was 50 nM.

**Figure 7:**
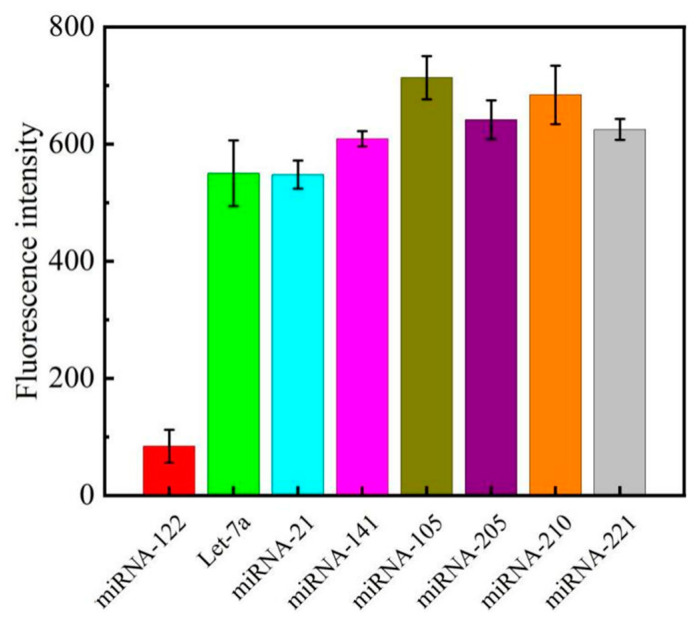
Relative fluorescence intensity at an emission wavelength of 340 nm versus different miRNA species, where the error bars represent the standard deviation of three replicate experiments.

The fluorescence spectra of various miRNA detectors are shown in Figure 7. It is obvious from the histogram that the fluorescence intensity of the target miRNA-122 is much lower after three testing conditions, which has a certain degree of differentiation compared with other miRNAs. It can be clearly seen in Figure S2 that, except for the low fluorescence value of miRNA-122, the fluorescence values of the miRNAs were high, indicating that it was difficult for other kinds of miRNAs to bind to DNA1 and undergo enzymatic reaction in the experiment; DNA1 was not hydrolysed, and fluorescent copper nanoclusters were successfully generated in the subsequent experiment. Taken together, this method has good discrimination ability for other homologous classes of miRNAs with good specificity and selectivity. Further, there was no significant difference in the standard curve of the four cycles, as shown in Figure S3, indicating the good reproducibility of the fluorescence signal-based miRNA detection system.

The capacity of a sensor to function in challenging settings, such blood serum, is one of its key metrics. To test the detection capabilities of the sensor, various concentrations of TD (1, 10, 100 nmol/L) were added to the serum solution for recovery tests, as listed in Table S2. The recovery (between 98.20% and 100.32%), relative standard deviation (RSD) (between 1.65% and 3.23%), and *p* < 0.05 are feasible. The results showed that the detection platform has potential advantages in the analysis and detection of complex biological samples.

## 4. Conclusions

In conclusion, we constructed a non-fluorescently labelled, low-cost DNA biosensor for sensitive detection of miR-122 down to 0.46 nM using Exo III and copper nanoclusters with fluorescent effects generated on the circular T structure of hairpin DNA1. The sensor is facilitated via target miRNA-122 hairpin DNA being released by Exo III water interpretation and separated from the reaction solution by the action of streptavidin immunomagnetic beads. Finally, the reaction solution tested showed essentially no fluorescent properties due to the failure of fluorescent copper nanocluster generation when copper ions were reduced without the action of background DNA long chain T. The first benefit of this approach is that it lowers the detection threshold by inadvertently discovering enzymatic circular amplification. Second, the DNA was created with fewer designed DNA strand segments, no fluorescent indicators, and simple testing procedures. Third, the DNA was designed without fluorescent markers, with fewer designed DNA strand segments and easy testing steps. Fourth, it can clearly distinguish the amount of miRNA content in the detection range compared with the experimental results without the addition of target miRNA-122. The test exhibits good selectivity, according to the experimental findings, and it can be utilised for qualitative analysis. This assay is highly feasible and has a strong chance of being widely used in disease prevention and treatment because it can also be used to detect other disease-related biomarkers by simply changing the experimental design’s target recognition sequence of DNA.

## Figures and Tables

**Table 1:** Comparison with some other fluorescence signal-based sensors for miRNA detection.

Detection Method	Linear Range	LOD	Reference
Enzyme-Assisted Amplification Strategies	10 fM–10 nM	4.7 fM	[35]
Fluorescent Silver Nanoclusters	0.2 nM–30 nM	0.1 nM	[36]
Catalytic Hairpin Assembly (CHA)	5 pM–0.5 nM	2.3 pM	[37]
Isothermal Cascade Amplification	2 aM–2 nM	0.82 aM	[38]
Exo III and Copper Nanocluster	5 nM–50 nM	0.46 nM	This work

## Data Availability

Not applicable.

## Supplementary Materials

The following supporting information can be downloaded at: https://www.mdpi.com/article/10.3390/bios13090854/s1, Figure S1. The TEM images of the nanoclusters before (A, B) and after (C) interaction with miR-NA. Figure S2. Experimental results Fluorescence values corresponding to various miRNAs. Figure S3. Comparison of the standard curves of the four cycles. Table S1. Synthesized Oligonucleotides sequences employed in this work. Table S2. The serum sample were tested for miRNA-211.
