# Supplementary material for: Enzyme-Assisted Amplification and Copper Nanocluster Fluorescence Signal-Based Method for miRNA-122 Detection
Source: Biosensors (Basel). 2023 Aug 28;13(9):854. doi: 10.3390/bios13090854 (PMC10526218; doi:10.3390/bios13090854)
Supplement: Supplementary file 1 [file biosensors-13-00854-s001.zip › biosensors-2529925-supplementary.pdf]

biosensors

MDPI

Supplementary

Enzyme-Assisted Amplification and Copper Nanocluster Fluorescence Signal-Based Method for miRNA-122 Detection

Yang Qing, Haobin Fang, Yuxing Yang, Yazhen Liao, Haiyu Li, Zhencui Wang and Jie Du *

State Key Laboratory of Marine Resource Utilization in South China Sea, College of Materials Science and Engineering, Hainan University, Haikou 570228, China; 20080500210023@hainanu.edu.cn (Y.Q.); 21220856000011@hainanu.edu.cn (H.F.); 20085600210065@hainanu.edu.cn (Y.Y.); 21220856000036@hainanu.edu.cn (Y.L.); 20080500110012@hainanu.edu.cn (H.L.); 20080500110014@hainanu.edu.cn (Z.W.)

* Correspondence: author: dujie@hainanu.edu.cn

1. Materials

Table S1. Synthesized Oligonucleotides sequences employed in this work.

Oligonucle-otide	Sequence（5'-3'）
miR-122	5'-UGGAGUGUGACAAUGGUGUUUG-3'
DNA1	5'-ATGGTGGGGTTTTTTTTTTTTTTTTTTTTTACCCCACCATTGTCACAC-TCCA-3'
DNA2	5'-CAAACACCATTTTTTTTTTTTTTTTTT-biotin-3'
DNA3	5'-ATGGTGTTTGTTTTTTTTTTTTTTTTTTTTT-3'

2. Image analysis

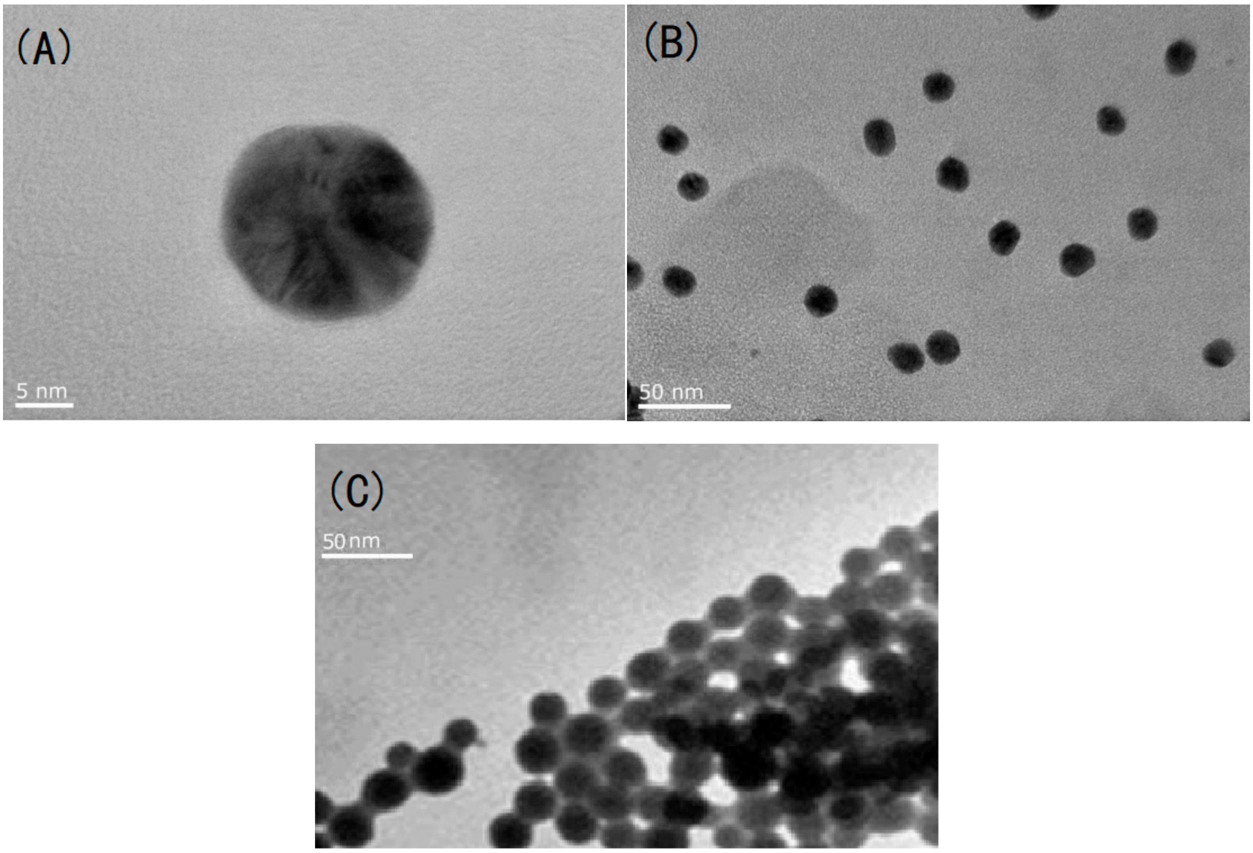

Figure S1. The TEM images of the nanoclusters before (A, B) and after (C) interaction with miRNA.

3. Specificity analysis

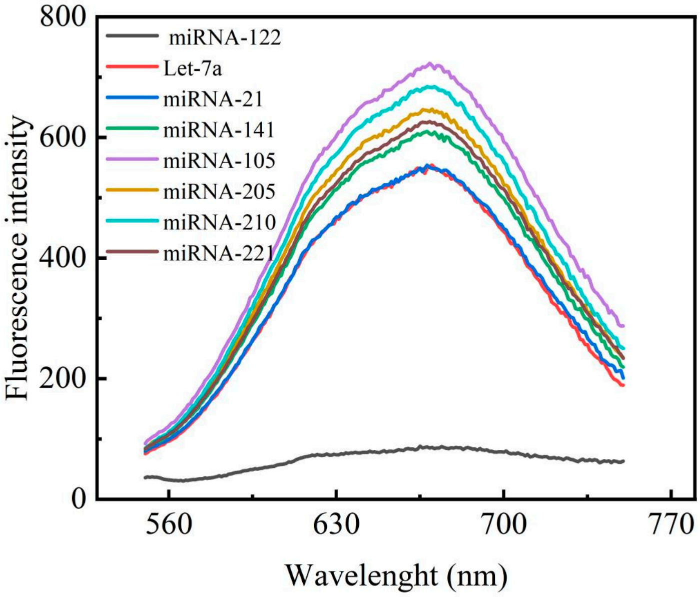

Figure S2. Experimental results Fluorescence values corresponding to various miRNAs.

4. Sensor specificity and reproducibility

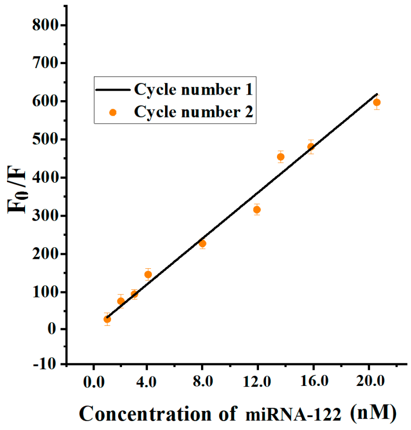

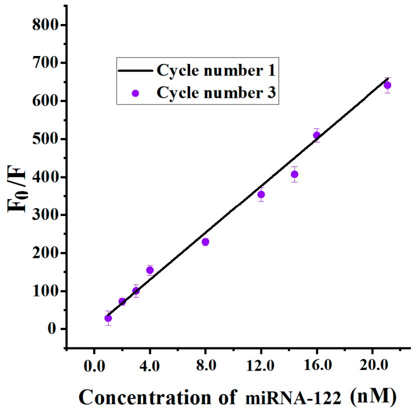

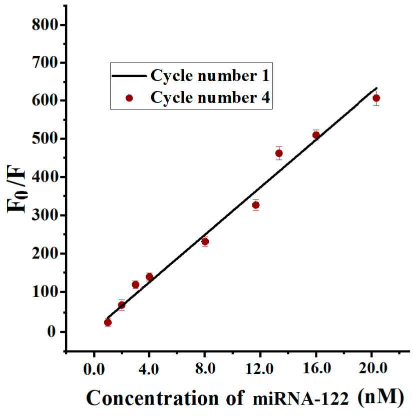

Figure S3. Comparison of the standard curves of the four cycles.

5. Detection in real sample

Table S2. The serum sample were tested for miRNA-211.

TD added(nmol/L)	Total found (nmol/L)	Recovery (%)	RSD (%)	p
1.0	0.982	98.20	1.65	0.02
10.0	9.975	99.75	3.23	0.04
100.0	100.323	100.32	1.95	0.03
